# Choroidal neovascularization secondary to punctate inner choroidopathy after resolution of multiple evanescent white dot syndrome: A case report

**DOI:** 10.1097/MD.0000000000047893

**Published:** 2026-03-06

**Authors:** Jie Bai, Zhicheng Li, Yanze Li, Yan Liu, Shan Wang

**Affiliations:** a Department of Ophthalmology, The Fourth Affiliated Hospital, Zhejiang University School of Medicine, Yiwu, Zhejiang, PR China; b Department of Ophthalmology and Otorhinolaryngology, Yiwu Second People’s Hospital,Yiwu, Zhejiang, PR China; c Department of Oral Pathology, School of Stomatology, Hainan Medical College, Haikou, Hainan, PR China.

**Keywords:** choroidal neovascularization, multiple evanescent white dot syndrome, optical coherence tomography, punctate inner choroidopathy, retina

## Abstract

**Rationale:**

White dot syndromes include multiple evanescent white dot syndrome (MEWDS) and punctate inner choroidopathy (PIC), which are rarely reported to coexist or occur sequentially in a single eye. The co-occurrence increases diagnostic complexity, and there are no previous reports of choroidal neovascularization (CNV) secondary to PIC following the resolution of MEWDS. This case report aims to supplement clinical evidence for the pathogenesis and treatment of such sequential ocular diseases.

**Patient concerns:**

A 42-year-old healthy female presented with blurred vision in her right eye for 3 days initially; 2 months later, her right eye vision worsened further, and 2.5 months after that, she developed blurred vision again with decreased visual acuity.

**Diagnoses:**

At the first visit, the patient was diagnosed with MEWDS in her right eye based on fundus examination, fundus autofluorescence, fundus fluorescein angiography, visual field test, and optical coherence tomography (OCT) findings. Two months later, she was diagnosed with PIC in the same eye due to new peripapillary yellow-white lesions and corresponding OCT changes. Another 2.5 months later, she was diagnosed with CNV secondary to PIC based on subretinal hemorrhage, abnormal vascular flow on OCT angiography, and fundus fluorescein angiography confirmation.

**Interventions:**

No treatment was given at the initial diagnosis of MEWDS. After the diagnosis of PIC, the patient received oral prednisone (1 mg/kg per day for 5 days, followed by gradual reduction over 6–8 weeks). For CNV secondary to PIC, intravitreal anti-vascular endothelial growth factor (VEGF) therapy (Ranibizumab, 0.5 mg/0.1 mL, once a month, twice in total) was administered.

**Outcomes:**

After 2 intravitreal anti-VEGF injections, the best-corrected visual acuity of the patient’s right eye improved to 20/20, fundic hemorrhage resolved, and OCT demonstrated complete resolution of CNV. During 2 years of follow-up, no recurrence of CNV or PIC was observed, and best-corrected visual acuity remained stable at 20/20.

**Lessons:**

MEWDS and PIC may share common etiological and pathogenetic mechanisms. PIC may develop sequentially after MEWDS in the same eye, and CNV may be a complication of PIC. A combination of oral corticosteroid therapy for PIC and timely intravitreal anti-VEGF injections for secondary CNV can achieve favorable long-term clinical outcomes. Regular follow-up is necessary for patients with MEWDS to monitor for potential progression to PIC and CNV.

## 1. Introduction

White dot syndromes (WDS) consist of a series of inflammatory eye diseases. While some experts consider each disease a distinct entity, others argue that WDS represent a spectrum of the same disease process.^[1]^ WDS do not always occur in isolation; this co-occurrence or sequential manifestation significantly increases the complexity of diagnosis. Multiple evanescent white dot syndrome (MEWDS) and punctate inner choroidopathy (PIC) are both classified under WDS, yet the coexistence of MEWDS and PIC in a single eye has been rarely reported. These 2 conditions exhibit different clinical courses but share overlapping clinical symptoms, which makes differentiation challenging. Both primarily affect young myopic women. MEWDS is characterized by multiple gray-white transient lesions throughout the fundus, and visual acuity typically improves as the condition resolves. In contrast, PIC is distinguished by multiple small yellow or white spots, which can occur either bilaterally or unilaterally at the posterior pole. Here, we present a case in which MEWDS preceded the sequential development of PIC and choroidal neovascularization (CNV) in the same eye.

## 2. Case history

**Figure 1.**
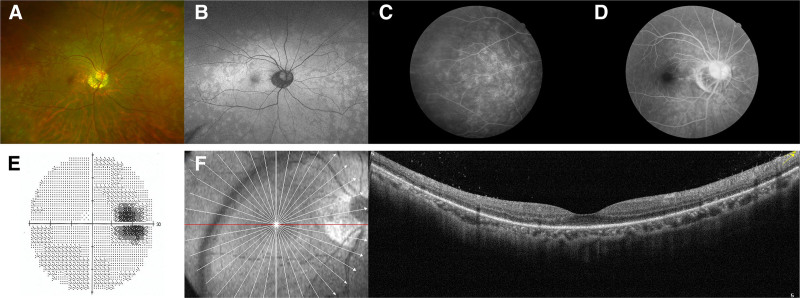
The right eye fundus detection. (A) At the first visit, color fundus photographs taken on the Optos platform show scattered gray-white lesions. (B) AF demonstrates hyperautofluorescence that corresponds to the gray-white lesions. (C and D) A late-phase FFA appears with hyperfluorescent dots and plaques and staining of the right optic nerve. (E) Visual field test showed enlargement of the blind spot. (F) The OCT shows a disrupted ellipsoid zone. AF = autofluorescence, FFA = fundus fluorescein angiography, OCT = optical coherence tomography.

**Figure 2.**
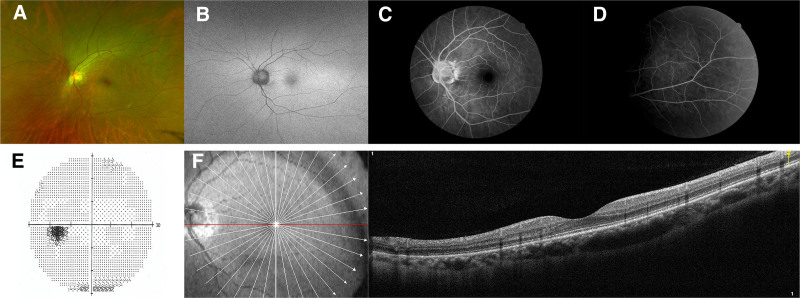
The left eye fundus detection. (A–F) Photographs, autofluorescence images, FFA, visual field, and OCT of the left fundus showing no noticeable changes. FFA = fundus fluorescein angiography, OCT = optical coherence tomography.

A 42-year-old woman, who has otherwise been in good health, presented with a complaint of experiencing blurred vision in her right eye for the past 3 days. Her refraction was −2.25 sphere in the right eye and −2.50 sphere in the left eye. The best-corrected visual acuity (BCVA) was 20/25 and 20/20 in the right eye and in the left eye, respectively. No abnormalities were found on slit lamp examination. Color fundus photograph revealed multiple gray-white spots in posterior periphery of the right eye (Fig. 1A). Fundus autofluorescence (AF) demonstrated hyperautofluorescent lesions (Fig. 1B). Fundus fluorescein angiography (FFA) showed hyperfluorescent dots and plaques corresponding to the white dots seen clinically (Fig. 1C) and upon staining of the right optic nerve during the late stage of the disease (Fig. 1D). Visual field test revealed an enlarged blind spot (Fig. 1E). Optical coherence tomography (OCT) demonstrated a disrupted ellipsoid zone (Fig. 1F). Examination of the left eye showed no noticeable funduscopic changes (Fig. 2A–F). The patient was then diagnosed with MEWDS. Laboratory testing, including complete blood count, erythrocyte sedimentation rates, C-reactive protein, complement C3, C4, and antinuclear antibodies were within normal ranges, serologic testing for syphilis, human immunodeficiency virus, leptospirosis, toxoplasmosis, and the herpes simplex virus type 2 (HSV-2) IgG antibody were negative; no evidence of sarcoidosis due to the presence of bilateral hilar lymphadenopathy on imaging of the chest. Since the evolution of MEWDS is normally limited, no treatment was suggested at that time of diagnosis.

**Figure 3.**
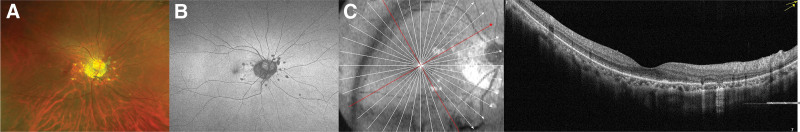
Description of fundus after 2 months. (A) Color fundus photographs taken 2 months after her first visit show multiple, small, round, yellow-white lesions around the disc of the right eye. (B) AF demonstrates multiple hypoautofluorescence lesions that corresponds to the yellow-white lesions around the disc. (C) The OCT shows irregular photoreceptor ellipsoid zone disruptions with underlying choroidal hyper-transmission. AF = autofluorescence, OCT = optical coherence tomography.

She returned 2 months later with worsening vision. The BCVA of the right eye decreased to 20/33, and fundoscopy showed multiple new, small, round yellow-white lesions situated peripapillary in the right eye (Fig. 3A). The AF demonstrated multiple hypoautofluorescence lesions surrounding the disc of the right eye (Fig. 3B). OCT showed irregular photoreceptor ellipsoid zone disruptions with underlying choroidal hyper-transmission (Fig. 3C). The patient was then diagnosed with PIC and received an oral prednisone course of 1 mg/kg per day for 5 days, followed by a gradual reduction within 6 to 8 weeks.

**Figure 4.**
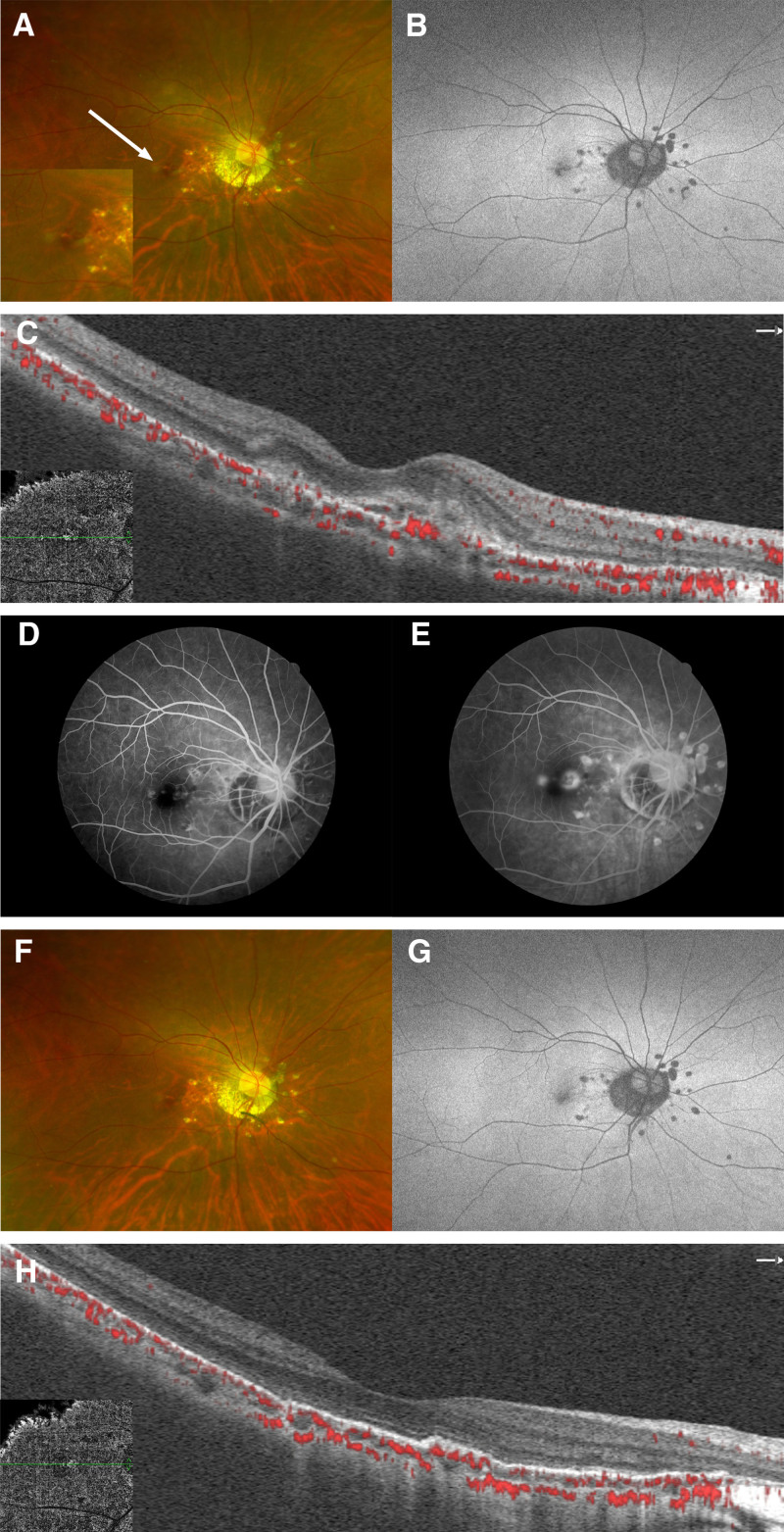
Description of fundus after CNV detected. (A) Two and a half months later, the color fundus of the right eye shows a hemorrhage in the fovea. (B) AF demonstrates hypoautofluorescence in the fovea, and PIC lesions surround the disc of the right eye, showing no notable changes compared to prior examinations. (C) OCTA demonstrates an abnormal vascular network. (D and E) CNV detected by FFA shows early hyperfluorescence and late leakage. (F–H) One month after her last intravitreal injection, fundus photography and OCTA of the right eye showed fibrous scarring of inactive peripapillary. AF = autofluorescence, CNV = choroidal neovascularization, FFA = fundus fluorescein angiography, OCTA = optical coherence tomography angiography, PIC = punctate inner choroidopathy.

**Figure 5.**
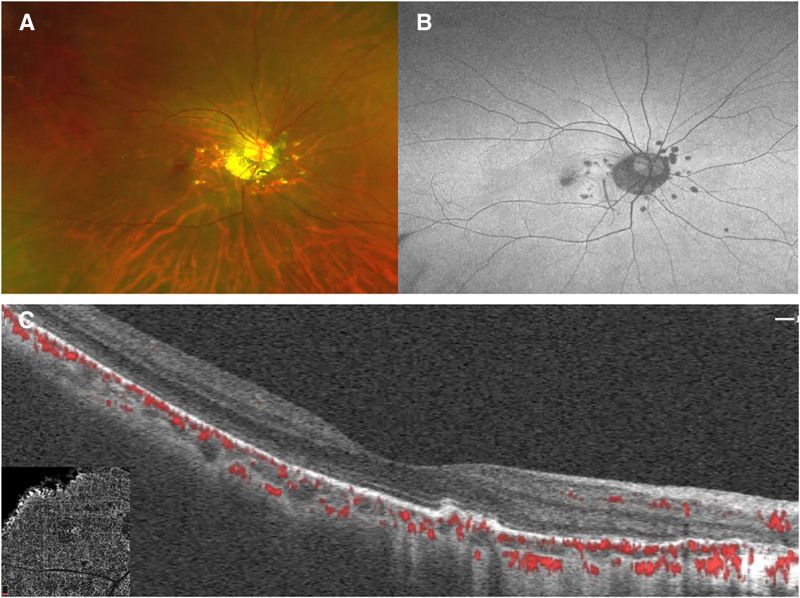
Description of fundus 2 years after the patient last intravitreal anti-VEGF injection. (A) Fundus photography at the final follow-up visit reveals no newly developed PIC lesions, with stable appearance of the posterior pole compared to prior imaging. (B) AF shows persistent hypoautofluorescence in the foveal region, consistent with the residual chorioretinal changes from previous disease activity. (C) OCTA demonstrates no detectable flow signals corresponding to choro.idal neovascularization, confirming complete remission of CNV. AF = autofluorescence, CNV = choroidal neovascularization, OCTA = optical coherence tomography angiography, PIC = punctate inner choroidopathy, VEGF = vascular endothelial growth factor.

However, 2 and a half months later, she again developed blurred vision, and her BCVA decreased to 20/50. A small fovea subretinal hemorrhage was discovered through fundus examination (Fig. 4A). The AF demonstrated hypoautofluorescence in the fovea (Fig. 4B). Optical coherence tomography angiography showed detectable flow above the retinal pigment epithelium (RPE) (Fig. 4C), and fundus fluorescein angiography confirmed the presence of CNV (Fig. 4D, E). The patient was then diagnosed with CNV secondary to PIC. Intravitreal anti-vascular endothelial growth factor (VEGF) (Ranibizumab, 0.5 mg/0.1 mL, once a month, twice in total) was administered for the treatment of CNV. One month later, the BCVA of the right eye improved to 20/20. The fundic hemorrhage had disappeared, and the OCT demonstrated CNV resolution (Fig. 4F–H). No recurrence of CNV or PIC was observed throughout the follow-up period. At the patient most recent visit, 2 years following her final intravitreal anti-VEGF injection, her BCVA was sustained at 20/20. Additionally, optical coherence tomography angiography examinations confirmed the absence of CNV recurrence, and fundus ealuations revealed no new PIC lesions, indicating long-term disease stability (Fig. 5).

## 3. Discussion

Symptoms related to WDS include blurred vision, visual field defects (such as an enlarged blind spot), and photosensitivity. Considering that MEWDS is a self-limiting condition with a favorable prognosis, spontaneous visual and anatomical recovery is the typical outcome for most patients, and treatment is usually not required.^[2]^ Although the lesion may spontaneously heal within 6 to 8 weeks,^[3,4]^ the development of peripapillary atrophy, choroidal neovascularization, and chorioretinal scars has been reported in some cases.^[5]^ In the present case, the patient developed PIC in the same eye approximately 2 months following the resolution of MEWDS. Concomitant with the onset of PIC, her visual acuity deteriorated, prompting the initiation of oral corticosteroid therapy to halt disease progression.

PIC typically occurs bilaterally; however, in this case, it was unilateral, and preceded by MEWDS.^[6]^ PIC is a type of idiopathic multifocal inflammatory chorioretinopathy, it is an immune-mediated disorder driven by genetic susceptibility and environmental triggers.^[7]^ Corticosteroids (CS) are key for PIC management:they inhibit immune/inflammatory pathways to control acute flares, have anti-angiogenic effects (induce PIC-associated CNV regression), possibly via reducing active CNV endothelial proliferation.^[8]^ Vienne-Jumeau et al conducted a 2-year retrospective longitudinal study on 36 PIC/MFC patients, analyzing corticosteroids (CS)’ impact on CNV. Results showed CS may help prevent CNV development and reduce recurrence in PIC/MFC.^[9]^

It is relatively easy to distinguish MEWDS from PIC. MEWDS presents as unilateral gray-white lesions at the RPE/retinal photoreceptor level, around the fovea and peripapillary regions, and typically does not leave scars or cause CNV.^[10]^ In our case, the patient PIC lesions occurred at the extrafoveal area, thus, achieving a better visual prognosis. The lesions of PIC are always located at the level of the RPE and choroid, typically deeper than those of MEWDS.

To our knowledge, no cases of CNV and PIC occurring in the same eye following a prior episode of MEWDS have been reported. However, there are studies that discuss the occurrence of secondary MEWDS following PIC.^[3,11]^ Due to the transient nature of MEWDS, histopathological evidence remains scarce. Consequently, most of our conclusions are primarily derived from clinical observations and interpretations of multimodal imaging findings. Serrar suggests that chorioretinal inflammation may precipitate secondary MEWDS, based on multimodal imaging which reveals inflammatory signs more frequently in secondary MEWDS cases.^[12]^ However, according to Meng study, aside from the extent of myopia, there were no significant differences in demographic, epidemiological, or clinical characteristics between primary and secondary MEWDS.^[13]^

There are many causes of CNV, which we now address by explicitly ruling out key potential causes in our patient. First, age-related macular degeneration (AMD) is excluded: the patient was 42 years old, well below AMD typical onset age (≥55 years), and fundus exam/OCT showed no drusen or geographic atrophy: hallmark features of AMD. Second, myopic CNV is not the cause: while the patient had mild right-eye myopia (−2.25D), myopic CNV typically arises in high myopia (>−6.0D) and is linked to posterior staphyloma, neither of which was present here. Third, infectious causes are ruled out, as laboratory tests for syphilis, human immunodeficiency virus, toxoplasmosis, and HSV-2 were all negative, eliminating infectious uveitis-related CNV. Finally, trauma or drug-induced CNV is excluded: the patient had no history of ocular trauma or use of CNV-associated medications (e.g., certain anti-cancer drugs).

The exact pathogenesis of inflammatory CNV in PIC and other uveitis-related diseases is not yet clear, but the ultimate common pathway includes the destruction of the RPE-Bruch complex and the release of VEGF leading to abnormal angiogenesis. CNV in PIC originates from inflammation-driven vascular dysfunction. Inflammatory damage to the RPE-Bruch membrane complex impairs the blood-retina barrier, which triggers the upregulation of VEGF, a key factor in driving abnormal angiogenesis. Specifically, following RPE-Bruch membrane disruption induced by MEWDS or PIC, chronic choroidal ischemia further upregulates VEGF expression, thereby promoting the formation of CNV.^[14]^

MEWDS and PIC are both types of uveitis associated with stress, systemic infections, or autoimmune diseases.^[15,16]^ In this particular case, the patient was considered to be in good health, having no previous history of eye diseases and did not take any medicine before. Additionally, both her physical examination and all laboratory tests conducted returned normal results. This case presents indirect evidence that MEWDS and PIC may share a common genetic susceptibility or environmental risk or that the syndromes may have a common etiologic or pathogenic background.

Development of PIC may occur in patient with MEWDS, suggesting that the conditions may share a common pathogenetic factor and/or genetic susceptibility. For CNV, early and timely anti-VEGF treatment can help achieve a good prognosis.

A limitation of our study is the lack of genetic testing and biomarker analysis (e.g., serum cytokines, VEGF levels), which prevented us from confirming shared etiological mechanisms between MEWDS and PIC. Notably, such investigations are not part of routine clinical care for WDS and were logistically unfeasible given our retrospective study design. However, recent research supports our hypothesis of potential shared pathogenesis: Meng et al^[10]^ reported overlapping genetic susceptibility (e.g., HLA-B27 association) and similar biomarker profiles (e.g., elevated serum VEGF) in MEWDS and PIC patients. We propose future prospective studies incorporate genetic and biomarker assessments. These could validate whether MEWDS and PIC share common etiological pathways, enhancing understanding of their progression and informing targeted interventions.

A further limitation of this study is its single-patient design. Given the unique clinical course of CNV secondary to PIC following the resolution of MEWDS in this case, our findings cannot be generalized to all MEWDS/PIC patients. However, single-case reports hold value in ophthalmology, as they facilitate the identification of rare disease sequelae and the generation of hypotheses for larger-scale studies. The sequential onset of MEWDS, PIC, and CNV in 1 eye, as described in this report, has not been previously documented, thereby expanding our understanding of the progression of WDS. We have referenced high-impact case reports on rare WDS complications to support the value of this observation. Future larger cohort studies should validate this disease trajectory and clarify the factors that predispose patients with MEWDS or PIC to develop CNV, which would aid in the development of evidence-based monitoring protocols and prophylaxis guidelines.

## Author contributions

**Conceptualization:** Jie Bai.

**Data curation:** Jie Bai.

**Formal analysis:** Jie Bai.

**Funding acquisition:** Jie Bai.

**Investigation:** Jie Bai, Zhicheng Li, Yanze Li, Yan Liu.

**Methodology:** Jie Bai, Zhicheng Li, Yanze Li, Yan Liu.

**Project administration:** Jie Bai.

**Resources:** Jie Bai.

**Software:** Jie Bai.

**Supervision:** Jie Bai, Shan Wang.

**Validation:** Jie Bai.

**Visualization:** Jie Bai.

**Writing – original draft:** Jie Bai.

**Writing – review & editing:** Jie Bai, Shan Wang.
